# Identification and validation of SUN modification-related anti-PD-1 immunotherapy-resistance signatures to predict prognosis and immune microenvironment status in glioblastoma

**DOI:** 10.1186/s12885-025-15345-9

**Published:** 2025-11-29

**Authors:** Hong Zhang, Meiyan Gao, Zhen Gao, Li Yao, Hong Sun, Huqing Wang, Ru Zhang, Shuqin Zhan

**Affiliations:** 1 Department of Neurology, The Second Affiliated Hospital of Xi’an Jiaotong University, No. 157, West Fifth Road, Xincheng District, Xi’an, 710004 China; 2 Department of Laboratory Medicine, Shaanxi Provincial Hospital of Traditional Chinese Medicine, Xi’an, 710003 China

**Keywords:** Glioblastoma, Posttranslational modification, SUMOylation, Ubiquitination, Neddylation, Immune microenvironment

## Abstract

**Background:**

Ubiquitination, SUMOylation, and neddylation (collectively termed SUN modifications) play crucial roles in cancer pathogenesis and immunotherapy resistance. This study investigated the prognostic significance of these modifications in glioblastoma (GBM).

**Methods:**

Key genes associated with SUN modifications and anti-PD-1 resistance were identified using integrated bioinformatic approaches, including differential expression analysis, Weighted Gene Co-expression Network Analysis (WGCNA), and machine learning algorithms. The expression levels of identified genes were subsequently validated in GBM cell lines using RT-qPCR and Western blotting. A prognostic risk model was constructed based on the key genes. Single-cell RNA sequencing (scRNA-seq) and spatial transcriptome analysis were further employed to characterize gene expression patterns.

**Results:**

Six prognostic genes (PLK2, CDC73, PSMC2, SOCS3, ETV4, and LMO7) were identified. CDC73, PSMC2, SOCS3, and ETV4 were upregulated, while PLK2 and LMO7 were downregulated in GBM cells. The six-gene prognostic risk model demonstrated excellent predictive performance, achieving an Area Under the Curve (AUC) exceeding 0.9. The derived risk score exhibited significant correlations with clinical features, immune infiltration levels, and drug sensitivity profiles. Furthermore, scRNA-seq and spatial transcriptome analysis revealed high SOCS3 expression specifically in monocytes and macrophages, suggesting its potential role in mediating the activity of these immune cells to influence tumor progression and drug sensitivity in GBM.

**Conclusion:**

This study established a robust six-gene prognostic model related to SUN modifications and anti-PD-1 therapy in GBM. The model demonstrates strong predictive ability and correlates with clinically relevant parameters, highlighting its potential utility for survival prediction and guiding therapeutic management decisions in GBM patients.

**Supplementary Information:**

The online version contains supplementary material available at 10.1186/s12885-025-15345-9.

## Introduction

Glioblastoma (GBM) commonly referred to the GBM (grade 4), which was updated to be glioblastoma, IDH-wildtype in the WHO 2021 CNS Tumor classification [1]. GBM and other malignant gliomas constitute the most prevalent and highly fatal brain tumors in the adult population, with an annual incidence of approximately 5.26 per million and about 17 thousand new diagnoses per year in the United States [2]. GBM has a dismal prognosis, with a median survival duration of approximately 12–18 months following diagnosis [3]. Current treatment for GBM includes surgical resection, radiotherapy, and chemotherapy; targeted or biologically directed approaches such as anti-VEGF therapy (e.g., bevacizumab) and multikinase inhibitors (e.g., regorafenib) have also been explored, although their benefits remain limited in unselected GBM [4]. Despite multimodal therapy, the 5-year overall survival for GBM remain below 5% [5]. GBM exhibits marked inter-tumor and intra-tumor heterogeneity, which drives therapy resistance through clonal evolution, regional variability in oncogenic signaling, and a diverse tumor immune microenvironment; these factors collectively hinder robust prognostication and durable treatment response [6]. Therefore, there is a pressing need to discover more efficacious and novel prognostic biomarkers for GBM in order to ameliorate its prognosis.

Posttranslational modifications (PTMs) are the biochemical modifications of proteins after protein biosynthesis, which control the protein abundance and function exceed inherent transcriptional regulation [7]. PTMs could modulate protein biosynthesis process via modification like phosphoryl, methyl, acetyl, and glycosyl. Recently, aberrant regulatory roles of PTMs in diseases are proposed. For example, USP36 has been demonstrated to promote tumorigenesis and drug resistance in GBM through deubiquitination [8]. Furthermore, targeting PTMs alter, a novel therapeutic method is also proposed. The latest study by Yue et al. demonstrated that suppressing lactylation in GBM could increase its sensitivity to cancer therapy [9]. Immune checkpoint blockade, particularly anti-programmed cell death protein-1 (PD-1)/PD-1 ligand-1 immunotherapy, exhibits considerable promise in the management of diverse cancer types, encompassing GBM [10]. However, still less than 10% of patients show an objective response to anti-PD-1 therapy [10]. PTM of PD-1 has been demonstrated to be a potential target for cancer immunotherapy, as it affects the anti-tumor immunity of T cells [11–13]. These evidences indicate the crucial role of PTMs in GBM progression and anti-PD-1 therapeutic research, prompting us to explore PTMs related biomarkers in GBM to predict the GBM occurrence and anti-PD-1 therapeutic efficiency.

Ubiquitination, alongside small ubiquitin-like modifier (SUMOylation) and neuronal precursor cell-expressed developmentally down-regulated protein 8 modification (neddylation), collectively constitute the three primary types of PTMs known as SUN [14]. Previous studies have highlighted the critical role of SUN in cancer cell apoptosis, the cell cycle, and other biological processes. For example, it is reported that the SUMOylation modification of HNRNPK at the specific site interferes its DNA-binding ability, and thus promotes GBM invasion [15]. In some studies, several SUN-related prognostic signatures based on SUN has been exploited, such as a two-gene SUMOylation signature in prostate cancer [16] and a three-gene ubiquitination signature in pancreatic ductal adenocarcinoma [17]. Nevertheless, a systematic investigation for SUN-related prognostic signature in GBM remains absent to date.

**Fig. 1:**
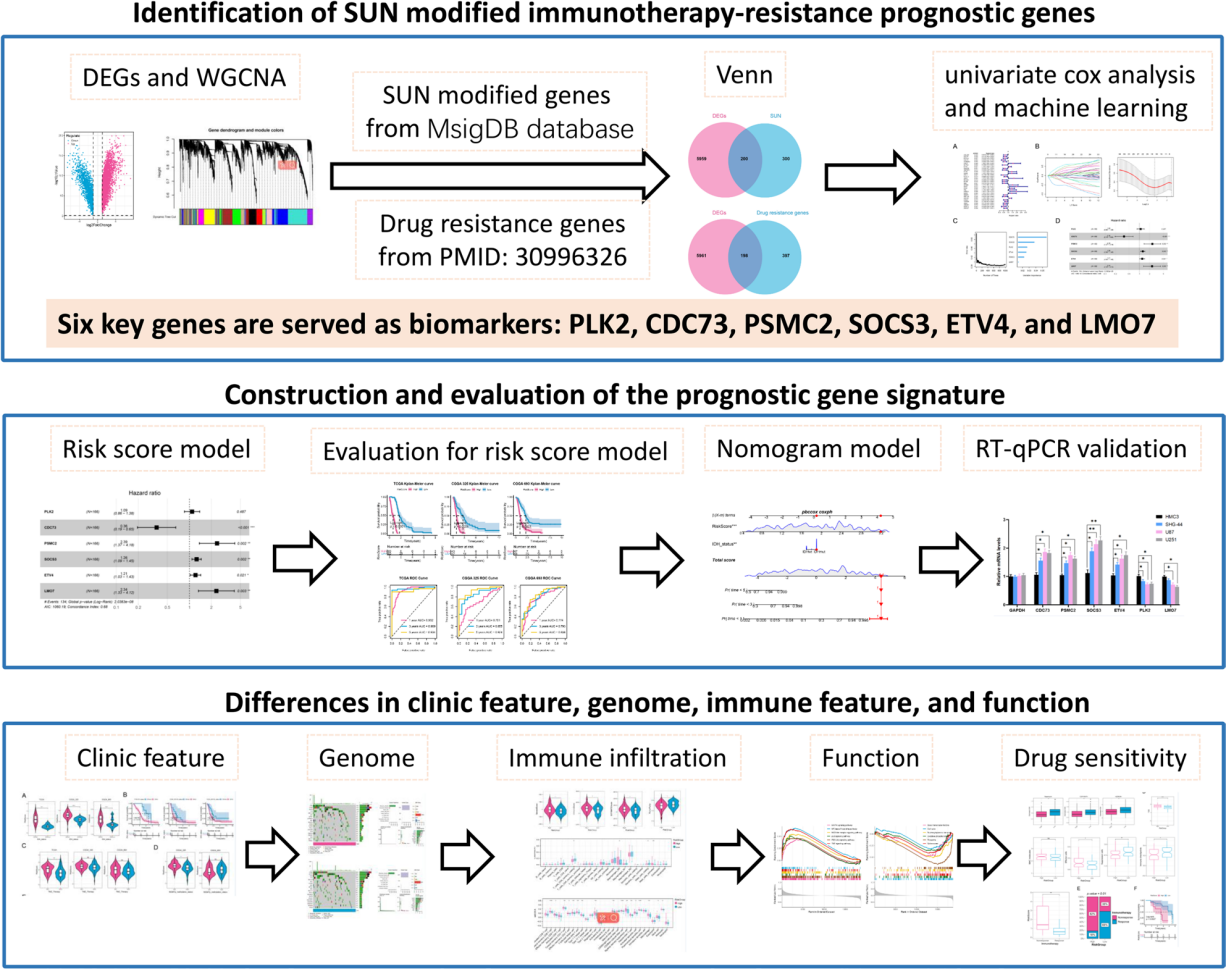
The flowchart of the study. DEGs, Differential expressed genes; SUN, ubiquitination, small ubiquitin-related modifiers (SUMOylation), and neddylation; WGCNA, Weighted gene co-expression network analysis

Considering the crucial role of SUN-related genes in the GBM development and antiPD-1 therapy, we hypotheses that there were key SUN genes which can be used for survival predicting and drug therapeutic outcomes in GBM. In our study, SUN modified anti-PD-1 immunotherapy resistance genes (SUNIRDEGs) were identified using bioinformatic tools through generating data from public database; and the main SUNIRDEGs were validated utilizing RT-qPCR and WB analysis. Following, a prognostic risk scoring system was developed utilizing the key genes, which might be efficient in predicting the disease and therapeutic outcomes. The research framework is depicted in Fig. 1.

## Methods

### Data source

The GBM data was retrieved from the Cancer Genome Atlas (TCGA)-GBM database [18] and China Glioma Genome Atlas (CGGA) database (CGGA-325 and CGGA-693 datasets). Then the human gene annotation files (GRCh38. P14) were retrieved from the GENCODE database [19]. Based on the annotation files, data from TCGA-GBM and CGGA database was preprocessing as listed below: (1) samples featuring missing or zero survival time were omitted; (2) samples were removed if they contained more than 50% missing values or unexpressed genes; (3) all expression values underwent log_2_(X + 1) transformation. Totally, 166 GBM samples were retrieved from the TCGA-GBM database; and 85 and 133 GBM samples were obtained from CGGA-325 and CGGA-693 datasets in CGGA database, respectively. Furthermore, RNA-sequencing data for 207 normal samples were downloaded from Genotype-Tissue Expression (GTEx) database.

Finally, Anti-PD-1 immunotherapy data GBM-PRJNA482620 were obtained from a previously literature [20], including17 patients with Nonresponse and 17 patients with Response.

### Differential expression analysis

Limma package [21] was adopted to select the differential expressed genes (DEGs) between GBM and normal tissues based on the 207 normal samples from GTEx database and 166 GBM samples from the TCGA-GBM database. For the consistency and standardization, all the data in the two databases were uniformly processed using the Toil process of UCSC XENA [22]. The criteria for selection were designed as False Discovery Rates (FDR) < 0.05 and |log_2_ fold change (FC)| >1.

### Weighted gene co-expression network analysis (WGCNA)

WGCNA analysis was utilized to determine the gene modules which were highly associated with GBM based on GBM-PRJNA482620 dataset by using WGCNA R package (v1.72) [23]. Firstly, after comprehensive consideration to ensure the robustness of the network, construction efficiency and biological significance, the top 5000 genes in GBM-PRJNA482620 were selected based on the mean absolute deviation (MAD). Next, WGCNA package was utilized to identify the gene modules which were highly linked to anti-PD-1 immunotherapy, with Nonresponse and Response as the phenotypic characteristics. In WGCNA, to minimize the potential impact of clinical confounding factors, we subsequently included gender and sex as covariates to investigate the correlations between each module and each covariate. The soft-thresholding power (β) was set to 6, ensuring a scale-free topology fit index (R²) >0.85. Genes with the top 50% variance were used to construct the co-expression network. Modules were identified using hierarchical clustering with a minimum module size of 30 and a dynamic tree-cut algorithm, followed by merging modules with eigengene correlation >0.75. Finally, the modules with correlation coefficient above 0.5 were selected as the key module, and the genes in the key modules were involved into function analysis to investigate their participated biological function and pathways.

### Identification of SUN modified immunotherapy-resistance genes

The SUN modified gene set was extracted from the Molecular Signatures Database (MsigDB) with the keywords “SUMOylation, ubiquitination, and neddylation”. The related pathway genes from REACTOME_SUMOYLATION, REACTOME_PROTEIN_UBIQUITINATION and REACTOME_NEDDYLATION were utilized as SUN modified genes for the subsequent analysis.

DEGs were intersecting with SUN modified genes and anti-PD-1 immunotherapy genes, namely DEGs-SUN genes and DEGs-drug resistance genes, respectively. Pearson correlation analysis was performed on the intersection results of the two parts of genes to identify SUN modified anti-PD-1 immunotherapy resistance genes (SUNIRDEGs), with the threshold of |R| >0.4 and *P* < 0.001.

### Identification of prognostic related genes

Based on the expression levels of above SUNIRDEGs in TCGA-GBM, and combined the clinical OS survival, univariate Cox was performed to evaluate the prognostic value of each gene utilizing survival package [24] in R software. *P* < 0.05 was deliberated as the threshold to filter genes with significant prognostic relevance.

### Exploitation and evaluation of the prognostic gene signature

Ten different machine learning algorithms [25] were performed to exploit the optimal predicting model. All the predicting models were obtained by LOOCV framework fitting based on TCGA-GBM data, and then these models were validated in CGGA-325 and CGGA-693 datasets. C-index was calculated to select the optimal models.

In accordance with the median value of the risk score, the cohort of patients with GBM was stratified into high-risk and low-risk groups. Following this, Kaplan-Meier survival analysis was conducted to assess the prognostic implications. Furthermore, both calibration and ROC curves were constructed to assess the prognostic impact of the risk score. Finally, to examine whether our prognostic risk score model possesses superior predictive effects, we also compared the SUNIRDEGs prognostic signature with 10 published signatures predicting patient outcomes [26]. For comprehensive comparison, the three datasets (TCGA-GBM, CGGA-325, and CGGA-693) were also combined into an integrated meta-cohort, which was used to evaluate the overall predictive performance of the risk score models across different populations.

### Construction of prognostic nomogram model

To ascertain the independent prognostic factors, Cox regression analyses, encompassing both univariate and multivariate approaches, were executed on clinical variables, specifically including the risk score, age, gender, Karnofsky Performance Status (KPS), and Methylguanine-DNA Methyltransferase promoter (MGMTp) methylation status. The clinical characteristics with significant correlation with clinical prognosis were selected (*P* < 0.05). The nomogram model was constructed utilizing the rms package (Version 6.8-0.8.8.8) [27] within the R software framework.

### The differences of genome, immune feature, and function

GBM mutation data were obtained from TCGA database for the subsequent analysis. The maftools package (version 2.17.0) [28] was adopted to select the mutation frequency of the top20 genes.

To further observe the correlation between risk score and immune infiltrating cells, three immune microenvironment analysis algorithms ESTIMATE [29], CIBERSORT [30], and ssGSEA [31] were utilized to evaluate the immune infiltration levels of each GBM sample.

Based on the corresponding subset h.all.v2023.2.Hs.symbols.gmt in MSigDB database, GSVA algorithm [32] was performed to estimate the hallmark enrichment score of all GBM samples to investigate the differential KEGG pathways between the risk groups.

### The differences of drug sensitivity and immunotherapy response

The sensitivity of each GBM samples to the chemotherapy drugs were evaluated based on the Genomics of Drug Sensitivity in Cancer (GDSC) database [33]. pRRophetic package (version 0.5) [34] in R software was utilized to quantify IC50.

TIDE [35] was utilized to calculate the sample immunotherapy response score. Furthermore, immunophenoscore (IPS) was utilized to calculate the scores of four different immunophenotypes: antigen presentation (MHC molecules), effector cells (EC), suppressor cells (SC), and immune checkpoints (CP) [36]. Finally, based on the anti-PD-1 immunotherapy data from GBM-PRJNA482620, the links between risk score and immunotherapy response was evaluated to predict the immunotherapy for patients with GBM.

### Key genes expression in various immune cells

Here, the single-cell RNA sequencing (scRNA-seq) data of GBM was retrieved from the GSE162631 dataset in Tumor Immune Signature Consortium Hub (TISCH2) database [37]. Combined with the spatial transcriptomics data from the spatial omics resource in cancer (SORC) [38], the expression levels of the key genes in different cells were evaluated. Quality control steps included excluding cells with < 200 or >6000 detected genes, cells with >10% mitochondrial gene expression, and doublets identified using the DoubletFinder package. Data were log-normalized and scaled before performing principal component analysis (PCA) and clustering using a shared nearest neighbor (SNN) modularity optimization algorithm with a resolution parameter of 0.6. Marker genes were identified using the Wilcoxon rank-sum test (adjusted *P* < 0.05).

### Identification of molecular subgroups of GBM

Based on the key gene expression data obtained, ConsensusClusterPlus package [39] in R software was utilized to conduct unsupervised clustering analysis to identify the molecular subgroups of GBM. The clustering cluster number was set as 2 to 9 k; the clustering method was chosen as KMeans algorithm; and the distance was calculated as Euclidean distance. At the same time, Kaplan-Meier curve was employed to acclimate to observe the survival of samples between subgroups.

### Cell culture

Cell lines, including Human brain astrocytes (SVG p12) and various human glioma cell lines (SHG44, U87, and U251), were obtained from the Cell Bank/Stem Cell Bank of the Chinese Academy of Sciences. These cells were subsequently cultivated in Dulbecco’s Modified Eagle’s Medium (DMEM) supplemented with 10% fetal bovine serum (FBS) and 1% penicillin-streptomycin.

### RT-qPCR

Total RNA was isolated using Trizol reagent (Invitrogen), adhering to the manufacturer’s guidelines. RNA concentration was accurately determined with a NanoDrop ND-1000 spectrophotometer (NanoDrop Technologies). Following this, reverse transcription was conducted to convert RNA into cDNA, employing SuperScript IV reverse transcriptase (Thermo Fisher Scientific, Waltham, MA, USA). For quantitative PCR (qPCR) analysis, Platinum™ Taq DNA Polymerase High Fidelity (Thermo Fisher Scientific) was utilized on an Applied Biosystems 7500 Real-Time PCR System (Applied Biosystems, Foster City, CA). The qPCR protocol encompassed an initial denaturation at 95 °C for 30 s, followed by 40 amplification cycles consisting of denaturation at 95 °C for 15 s, primer annealing at 60 °C for 30 s, and DNA extension at 75 °C for 60 s. The primer sequences used for qPCR analysis are listed in Table 1, and gene expression levels were normalized to glyceraldehyde-3-phosphate dehydrogenase (GAPDH) as an internal reference.

**Table 1:** The primers

Primers	Primer sequence (5’ to 3’)	Length (bp)
GAPDH-S	GGAAGCTTGTCATCAATGGAAATC	168
GAPDH-A	TGATGACCCTTTTGGCTCCC	
CDC73-S	TGACACTGAAATCTGTAACGGAGG	104
CDC73-A	ATTTGGGGGAGGTCTTGCTT	
PLK2-S	GCAGTAGAAGGTCAATGGCTCA	84
PLK2-A	CAATCTGCCTGAGGTAGTATCGAAC	
PSMC2-S	GTATTAAAGAATCTGACACTGGCCT	153
PSMC2-A	CGTTGATAATGTATTTTGGGTCCTC	
SOCS3-S	CCTACTGAACCCTCCTCCGA	242
SOCS3-A	TGGTCCAGGAACTCCCGAAT	
LMO7-S	GCCCACAGGATTCTATGCTTCTT	242
LMO7-A	TCCCACTGACTGACCTGTTACG	
ETV4-S	AAGGAGACATCAAGCAGGAAGG	180
ETV4-A	CGACCTCCTCAGGCTCAATG	

### Western blotting (WB)

WB was used for detection of protein levels of the key genes (PLK2, CDC73, PSMC2, SOCS3, ETV4, and LMO7). Total protein was isolated using RIPA lysis buffer, and then the protein concentration was quantified using a bicinchoninic acid (BCA) kit. The membranes were incubated with primary antibodies at 4 °C overnight, followed by incubation with second antibody. The primary antibodies used in this assay were as follows: anti- PLK2 Antibody (1: 1000, 15956-1-AP, Proteintech Group, Wuhan, China), anti- CDC73 Antibody (1: 1000, 66490-1-Ig, Proteintech Group, Wuhan, China), anti- PSMC2 Antibody (1: 1000, GB113120, Servicebio, Wuhan, China), anti- SOCS3 Antibody (1: 1000, GB113792, Servicebio, Wuhan, China), anti- ETV4 Antibody (1: 1000, 10684-1-AP, Proteintech Group, Wuhan, China), anti- LMO7Antibody (1: 1000, 29392-1-AP, Proteintech Group, Wuhan, China), anti- GAPDH Antibody (1: 1000, GB15004, Servicebio, Wuhan, China), The second antibody used in this assay were HRP goat anti-rabbit (1: 3000, GB23303, Servicebio, Wuhan, China) and HRP goat anti-mouse (1: 3000, GB23301, Servicebio, Wuhan, China). Protein quantification normalization was established utilizing GAPDH as the endogenous reference. Immunoreactive bands were subsequently detected through enhanced chemiluminescence substrates (ECL, Sigma-Aldrich) and subjected to densitometric quantification employing ImageJ software suite (v1.8.0, NIH, USA).

### Statistical analysis

Statistical analyses were conducted using R software (version 4.3.3). To compare differences between the two groups, the Wilcoxon rank-sum test was applied. Correlations between the groups were assessed using the Spearman correlation method. The Cox regression results were tested by proportional hazards (PH) assumption. A *P*-value less than 0.05 was viewed statistically significant.

## Results

### Identification of SUN modified immunotherapy-resistance genes

**Fig. 2:**
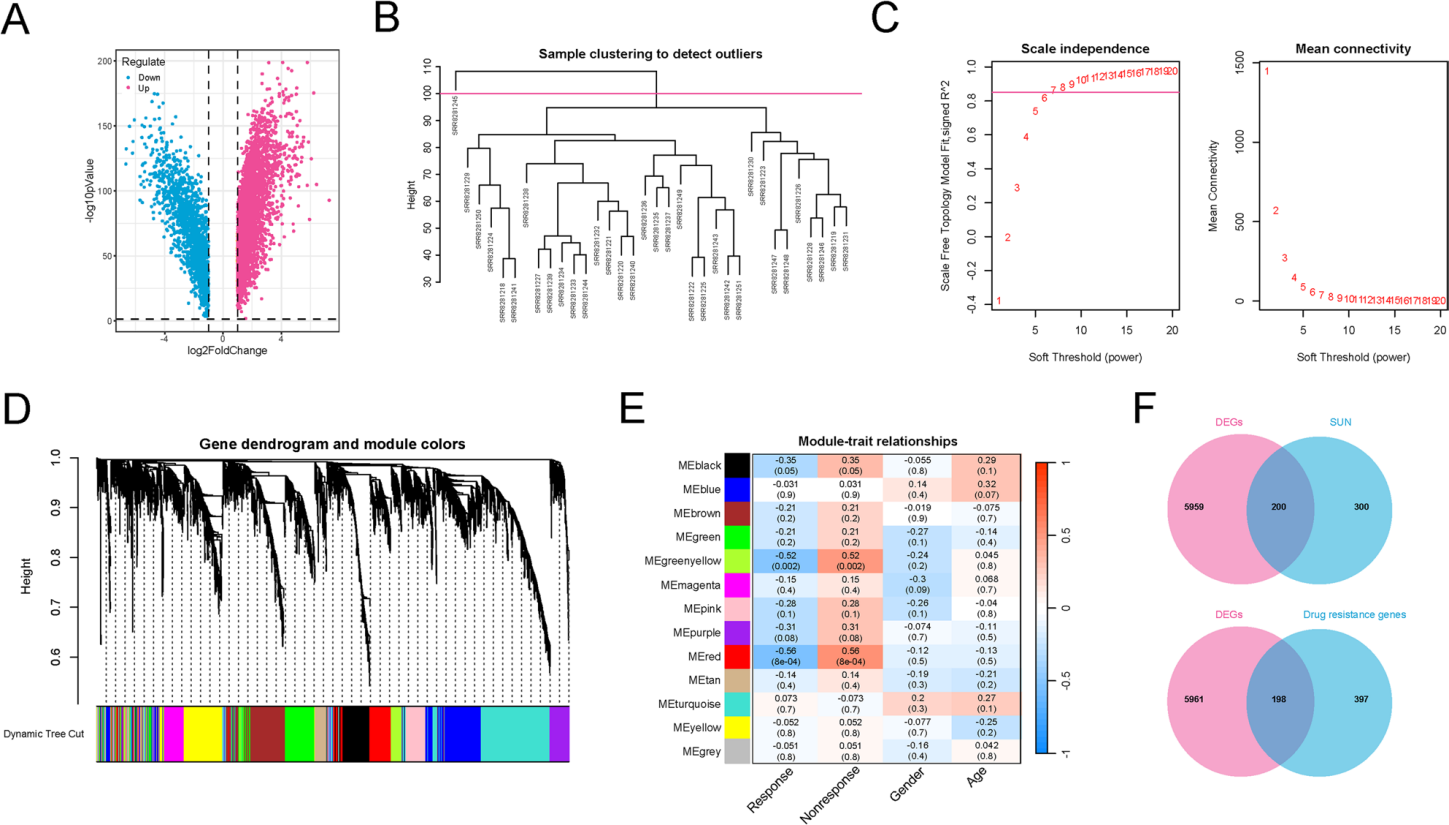
Identification of SUN modified immunotherapy-resistance genes. **A** Volcano of the differential expressed genes DEGs between GBM and control groups. **B** Sample level clustering. **C** Scale-free soft threshold distribution. The horizontal axis represents the weight parameter power value; the vertical axis of the left panel is Scale Free Topology Model Fit, that is, signed R^2. The higher the square of the correlation coefficient, the closer the network is to the scale-free distribution; the vertical axis of the right panel represents the mean of all adjacency functions. **D** Module clustering tree. Different colors represent different modules. **E** Heat map of correlation between modules and clinical traits. The vertical axis represents different modules; the horizontal axis represents different traits; and each square represents the correlation coefficient and P value. **F** Venn analysis

In differential expression analysis, a total of 6159 DEGs between GBM (from the TCGA-GBM dataset) and normal brain tissues (from the GTEx dataset) were determined (FDR < 0.05 and |log_2_FC| >1), with 4381 upregulated, and 1777 downregulated (Fig. 2A). In the WGCNA, the anti–PD-1 immunotherapy dataset (GBM–PRJNA482620) was used to identify gene modules associated with treatment response; and hierarchical clustering displayed that SRR8281245 sample was clearly deviant (Fig. 2B), which were deleted in the following analysis. Appropriate soft threshold was chosen as 7 and all the GBM samples were divided into 13 modules according to the dynamic tree cutting algorithm (Fig. 2C-D). Following, green-yellow and red modules showed the strongest positively correlated with immunotherapy, with correlation coefficient above 0.5 (Fig. 2E). Markedly, the green-yellow and the red module genes both participate in the same pathways such as RNA transcription modification, ubiquitin-mediated proteolysis, peroxisome, longevity-regulating pathway, and peroxisome (Figure S1), indicating that the genes in green-yellow and red modules exhibited the same phenotypic functions. Hence, the 595 genes included in the two modules were served as the immunotherapy-resistance-related genes. After intersecting analysis, a total of 200 overlapping DEGs-SUN and 198 DEGs-drug resistance genes were obtained (Fig. 2F). Finally, the correlations between 200 intersecting DEGs-SUN and 198 DEGs-drug resistance genes were conducted using a Pearson method, and genes with correlation coefficient above 0.4 and *P* < 0.001 were considered as the cross-talk genes (Table S1). After integration and deduplication, 355 cross-linked genes were obtained as SUNIRDEGs.

### Exploitation of the prognostic gene signature

**Fig. 3:**
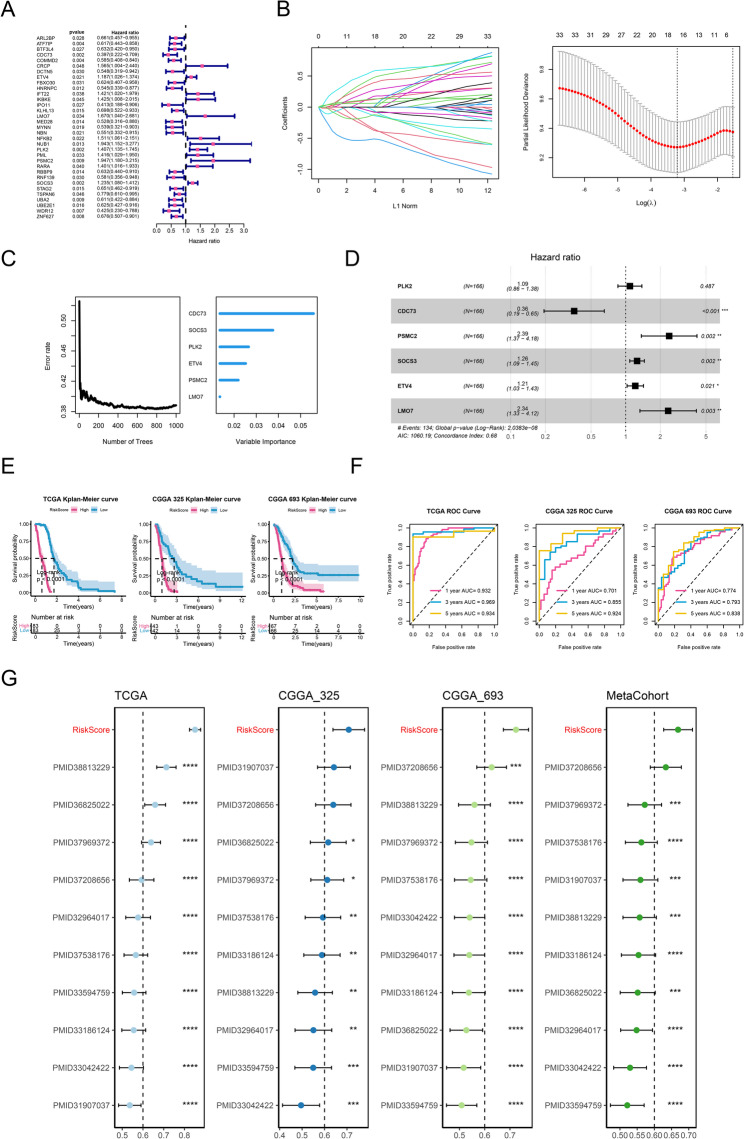
Exploitation of the prognostic gene signature. **A** Univariate Cox analysis for selection of prognostic genes. **B** LASSO analysis. Left panel represents the coefficients. Right panel represents the selection of λ. The two dashed lines indicate two special values of λ: lambda.min on the left and lambda.1se on the right. **C** RSF algorithm to determine the number of gene number with the least error, and the importance of the six most valuable gene feature. **D** Univariate Cox analysis for the six key genes to construct the risk score model. **E** Kaplan-Meier analysis for the risk score. **F** Receiver operator characteristic (ROC) to evaluate the effectiveness of risk score model. **G** Comparation for the signature in our results and the ten publications, showing in C index. *, *P* < 0.05; **, *P* < 0.01. ***, *P* < 0.001. ****, *P* < 0.0001

All 355 SUNIRDEGs were subjected to univariate Cox regression analysis using the TCGA-GBM cohort, and the prognostic performance of the resulting models was subsequently validated in the CGGA-325 and CGGA-693 cohorts. Finally, 33 prognostic SUNIRDEGs were confirmed to be markedly correlated with the overall survival (OS) (Fig. 3A, Table S2). Then, based on the 33 prognostic SUNIRDEGs, 10 algorithms were performed to select an optimal algorithm to exploit the prognostic signature. The results demonstrated that lasso + RSF exhibited best performance in prognosis in all the three datasets, with highest average C-index as 0.761 (Figure S2). Finally, Lasso identified 16 key genes, and the following RSF analysis confirmed six key signature genes: PLK2, CDC73, PSMC2, SOCS3, ETV4, and LMO7 (Fig. 3B-D). Based on the six key signature genes, the risk score model was formulated as the prognostic signature.

### Evaluation of the prognostic gene signature

in the TCGA-GBM dataset, all GBM samples were categorized into high- and low-risk cohorts based on the median risk score. Kaplan-Meier survival analysis demonstrated that patients in the high-risk cohort had noticeably worse OS compared to those in the low-risk cohort (Fig. 3E, *P* < 0.0001). The ROC curve exerted that the predicting ability of the risk score model were excellent, with AUC above 0.9 (Fig. 3F). The predicting ability of the risk score model were confirmed both in CGGA-325 and CGGA-693 datasets (Fig. 3E and F). To further evaluate the predictive performance of risk score, its predictive power was compared with signatures in ten previously reported literatures (the information of the ten literatures is listed in Table S3). The ten signatures were mainly derived from distinct biological mechanisms, including immune regulation, cell death–related genes, or metabolism-associated signatures; and none of which were directly related to SUN modification. Our results illustrated that our risk score model had better predictive performance than almost all the signatures in each dataset (including metacohort of TCGA-GBM, CGGA-325, and CGGA-693 dataset) (Fig. 3G). These results comprehensively implied that the risk score model related to SUN modified immunotherapy-resistance genes in our study displayed fantastic predicting ability in OS of patients with GBM and might be a robust signature in GBM prognosis.

### Construction of prognostic nomogram model

**Fig. 4:**
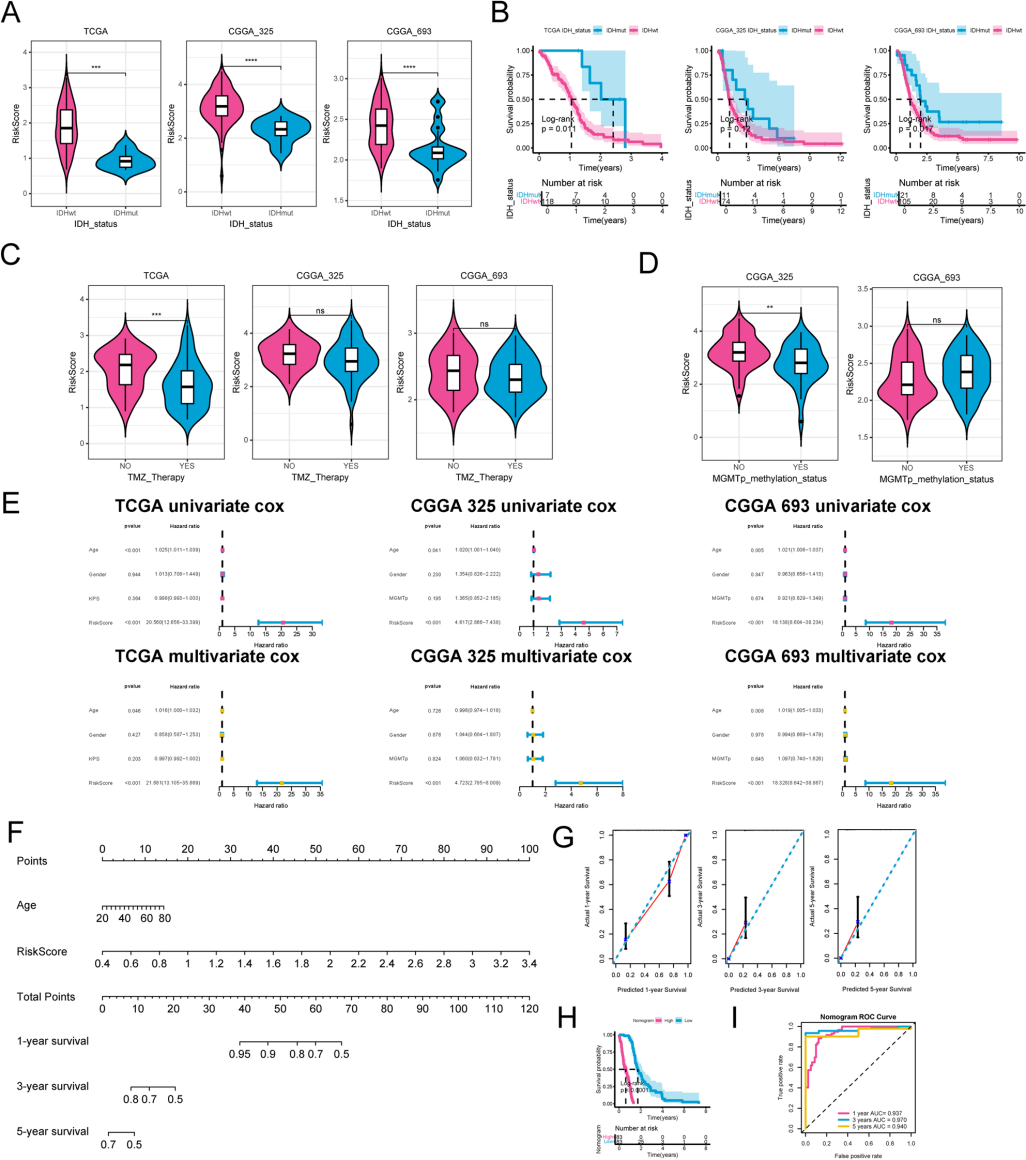
Correlation of the risk score with clinic features and construction of nomogram model. **A** Differences of risk score between IDHwt and IDHmut groups. **B** Kaplan-Meier analysis between IDHwt and IDHmut groups. **C** Differences of risk score between in patients with or without TMZ therapy. **D** Differences of risk score between different MGMTp methylation status. **E** Univariate and multivariate Cox correlation analysis for selection of the independent prognostic factors. **F** Construction of the nomogram model. **G** Calibration curve for evaluation of the nomogram model. **H** Kaplan-Meier analysis for different nomogram score. **I** The ROC curve for evaluation of the nomogram model. ns, not significant; *, *P* < 0.05; **, *P* < 0.01. ***, *P* < 0.001. ****, *P* < 0.0001

The clinical analysis suggested substantially different risk score between different IDH statues, showing as higher risk score in IDH wild-type (IDHwt) group than that in IDH mutation (IDHmut) group (Fig. 4A, *P* < 0.001). Kaplan-Meier analysis displayed that IDHwt was correlated with shorter survival time (Fig. 4B). Postoperative temozolomide (TMZ) combined with chemoradiotherapy is the first choice for therapy of GBM [40]. Our findings demonstrated that risk score was higher in patients who did not receive TMZ therapy (Fig. 4C). MGMTp methylation status can predict the sensitivity of patients with GBM to TMZ therapy [41]. In our results, patients who did not develop MGMTp methylation had a higher risk score (Fig. 4D). These results indicated that risk score was obviously associated with the clinic features of patients with GBM and might be an effective indicator in clinic.

Considering that WHO CNS5 criteria defined IDH-wildtype (IDHwt) as GBM, all IDHwt samples were involved into the Cox and nomogram model construction. In the subsequent analysis, univariate and multivariate Cox analysis implied that risk score were independent predictive factors in GBM (Fig. 4E and Table S2). The independent factors risk score were further involved into the nomogram (Fig. 4F). The calibration curve implied that the gradients between measured OS and nomogram-predicted OS were close to 1 (Fig. 4G), thereby demonstrating the efficacy of the nomogram. And the Kaplan-Meier survival curve revealed an inverse correlation between the nomogram score and survival probability (Fig. 4H). Furthermore, the ROC curve also indicated the robust predictive capacity of nomogram in OS, with AUC above 0.9 (Fig. 4I).

### The differences of genome, immune feature, and function

**Fig. 5:**
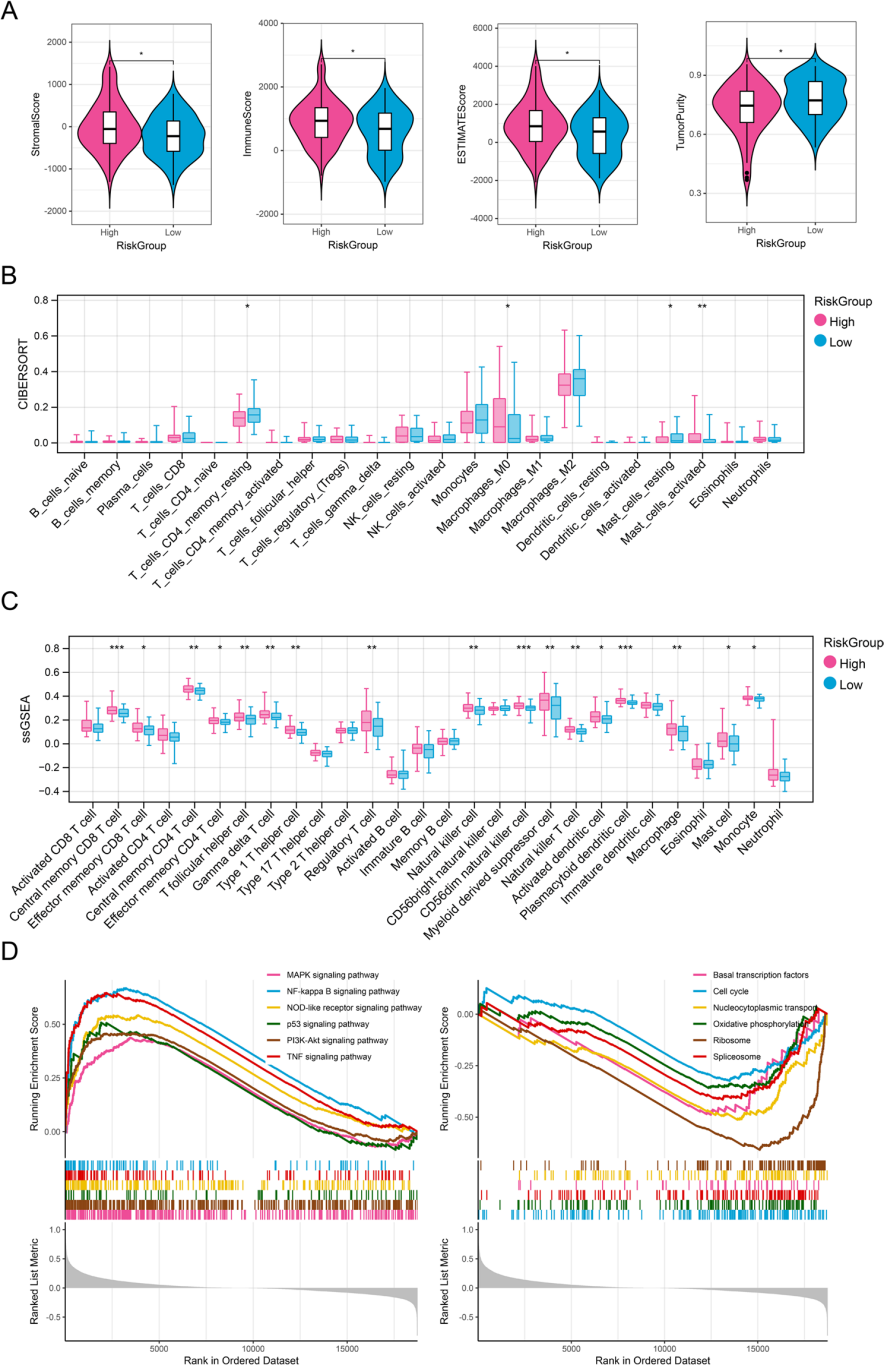
The differences of immune feature, and function between different GBM groups. **A** ESTIMATE algorithm for investigation of differences of stromal score, immune score, ESTIMATE score, and tumor purity between different risk groups. **B** CIBERSORT algorithm for investigation of differences of immune cells between high- and low-risk groups. **C** ssGSEA algorithm for investigation of the differences of immune cells between high- and low-risk groups. **D** The differences of KEGG pathways between high-and low-risk groups (www.kegg.jp/kegg/kegg1.html). ns, not significant; *, *P* < 0.05; **, *P* < 0.01. ***, *P* < 0.001. ****, *P* < 0.0001

To further elucidate the biological differences associated with the risk score, we analyzed gene mutation profiles and immune microenvironment characteristics between the high- and low-risk groups. Gene mutation occurred in both high- and low-risk groups and the mutation frequency type mainly was missense mutation and SNP (Figure S3). The ESTIMATE analysis revealed significant differences in immune, stromal, ESTIMATE scores, and tumor purity between the two risk groups, with the high-risk group exhibiting elevated immune, stromal, and ESTIMATE scores, as well as reduced tumor purity (Fig. 5A, *P* < 0.05), indicating active immune response under high-risk score. Moreover, immune cell infiltration levels were also investigated utilizing CIBERSORT and ssGSEA (Fig. 5B and C). The analysis revealed significant variations in immune infiltration levels between the two risk groups, with the high-risk group exhibiting elevated levels of regulatory T cells and natural killer cells compared to the low-risk group (Fig. 5C, *P* < 0.01). Functional annotation indicated that the pathways distinguishing the two risk groups were primarily associated with oncogenic processes, including the MAPK, NF-kappa B, NOD-like receptor, p53, and PI3K-Akt signaling pathways (Fig. 5D).

### The differences of drug sensitivity and immunotherapy response

**Fig. 6:**
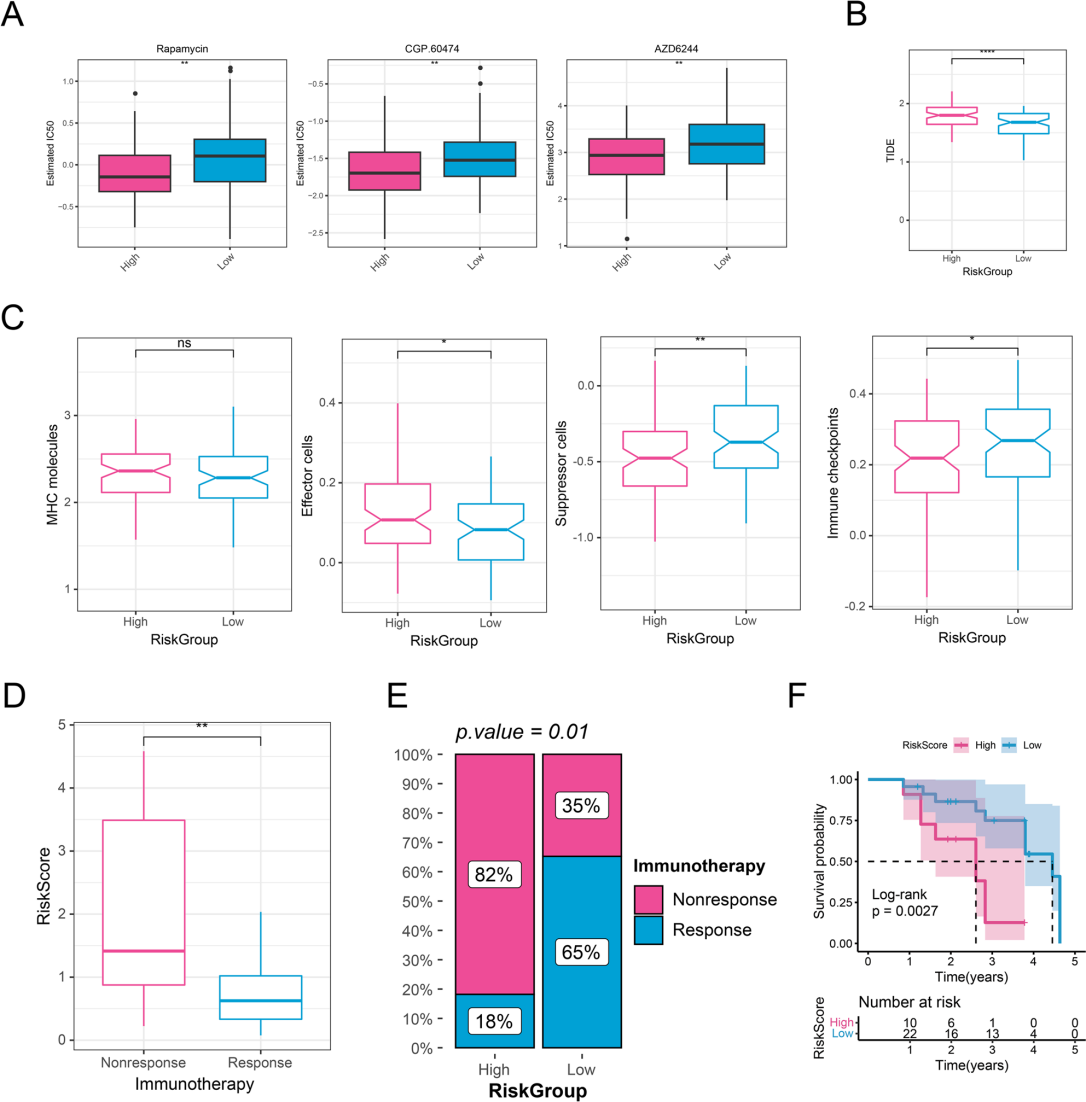
The differences of drug sensitivity and immunotherapy response between different GBM groups. **A** The differences of IC50 of drugs between high-and low-risk groups. **B** The differences of TIDE between high-and low-risk groups. **C** The differences of IPS score between high-and low-risk groups. **D** The differences of risk score between different immunotherapy groups. **E** The distribution of the patients with response or non-response to ICB treatment between high- and low-risk groups. **F** Kaplan-Meier analysis. ns, not significant; *, *P* < 0.05; **, *P* < 0.01. ***, *P* < 0.001. ****, *P* < 0.0001

Drug sensitivity analysis indicated significant differences of 25 drugs in IC50 values between the two risk groups. Notably, Rapamycin, CGP.60,474, and AZD6244 exhibited substantially higher IC50 values in the low-risk group compared to the high-risk group (Fig. 6A, *P* < 0.01), indicating reduced drug sensitivity in the high-risk cohort. Furthermore, the high-risk group had a significantly higher TIDE score than the low-risk group (Fig. 6B, *P* < 0.0001), implying that GBM in the high-risk group may exhibit enhanced immune evasion and a diminished response to ICB therapy. Additionally, IPS analysis displayed that suppressor cells (SC) and immune checkpoints (CP) were significantly more abundant in the low-risk group compared to the high-risk group (Fig. 6C, *P* < 0.05), implying potential sensitivity to ICB treatment in the low-risk cohort. Moreover, the risk score was markedly higher in the Nonresponse group compared to the Response group (Fig. 6D, *P* < 0.01) and high-risk group contained more Nonresponse patients (Fig. 6E, *P* < 0.05). High risk score was substantially linked to poor prognosis (Fig. 6F, *P* < 0.01).

###  The expression validation of the key genes in glioblastoma

**Fig. 7:**
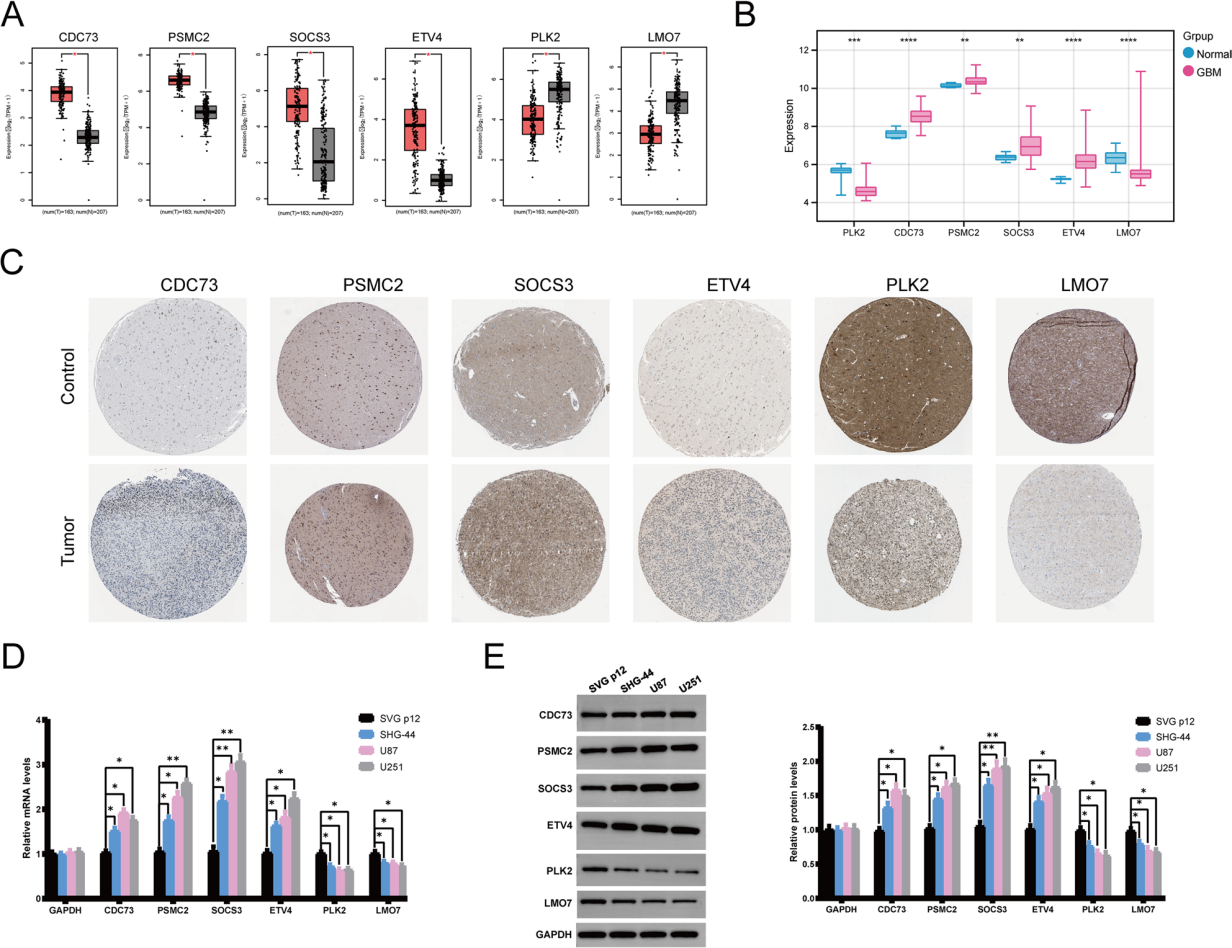
The expression validation of the key genes in glioblastoma. **A** The expression of CDC73, PSMC2, SOCS3, ETV4, PLK2, and LMO7 between control and tumor based on TCGA-GBM data in the GEPIA. **B** The expression of CDC73, PSMC2, SOCS3, ETV4, PLK2, and LMO7 between control and tumor in the GSE16011 dataset from GEO database. **C** The expression of CDC73, PSMC2, SOCS3, ETV4, PLK2, and LMO7 between control and tumor in the HPA database (https://www.proteinatlas.org/), the control group: histologically normal cerebral cortex tissues, the tumor group: pathologically confirmed glioma specimens. **D** The expression levels of CDC73, PSMC2, SOCS3, ETV4, PLK2, and LMO7 in human glioblastoma cells (SHG-44, U87, and U251) and control human astrocytes (SVG p12) detected by RT-qPCR analysis. **E** The expression levels of CDC73, PSMC2, SOCS3, ETV4, PLK2, and LMO7 in human glioblastoma cells (SHG-44, U87, and U251) and control human astrocytes (SVG p12) detected by western blotting analysis. *, *P* < 0.05; **, *P* < 0.01; ***, *P* < 0.001; ****, *P* < 0.0001

The expression levels of the six identified SUN modified immunotherapy resistance genes were investigated in the TCGA-GBM (via Gene Expression Profiling Interactive Analysis (GEPIA) database), Gene Expression Omnibus (GEO, GSE16011 dataset), and Human Protein Atlas database (HPA) databases. The results indicated that CDC73, PSMC2, SOCS3, and ETV4 were substantially upregulated in tumor group than that in control group, whereas PLK2 and LMO7 were substantially downregulated in tumor group than that in control group (Fig. 7A-C). RT-qPCR and WB validation also validated that CDC73, PSMC2, SOCS3, and ETV4 were substantially upregulated in GBM cells (SHG-44, U87, and U251) than that in control astrocytes group (SVG p12), whereas PLK2 and LMO7 were substantially downregulated in GBM cells than that in control microglia (Fig. 7D and E and S4, *P* < 0.05).

### The expression of the key genes in various immune cells

**Fig. 8:**
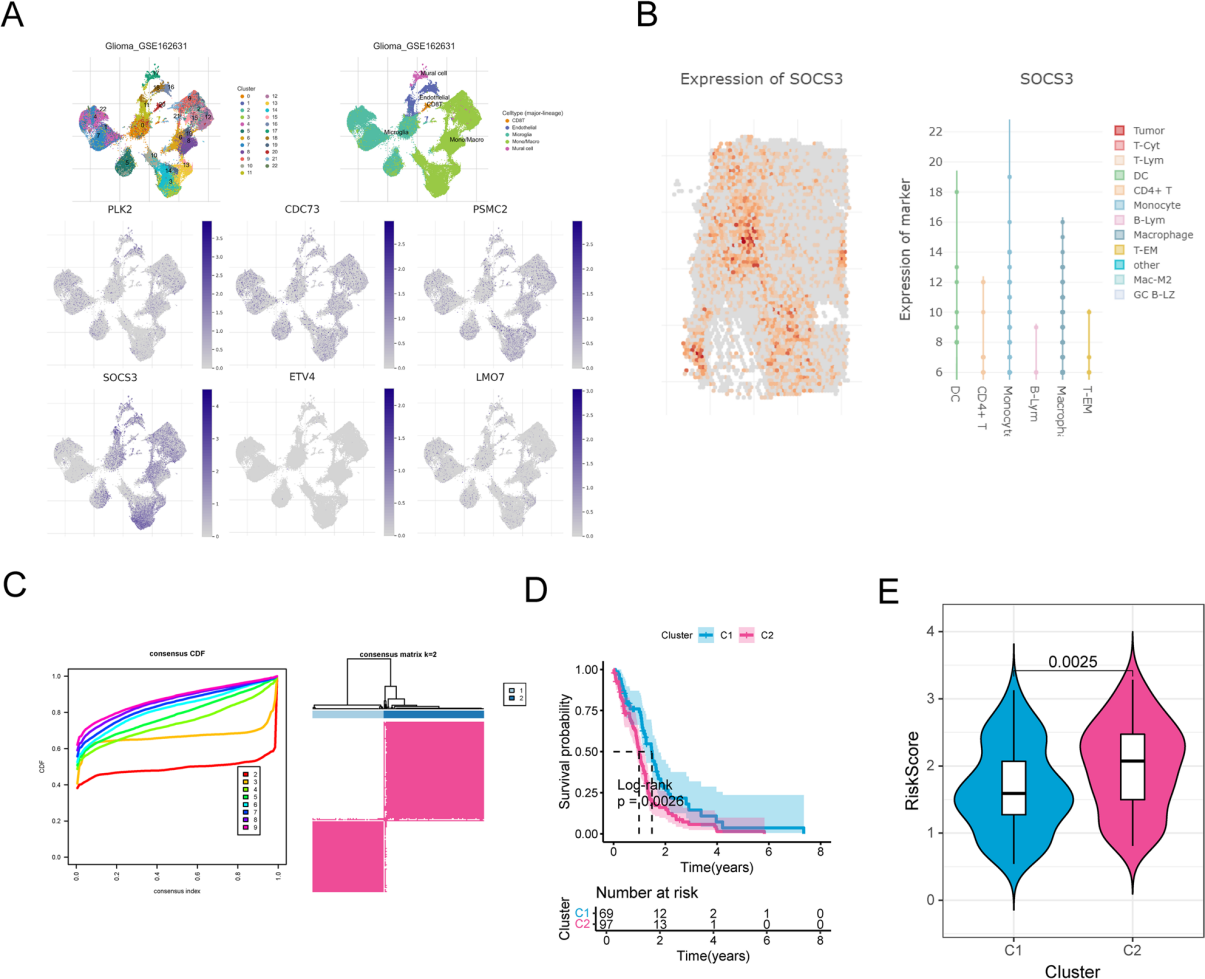
The expression of the key genes in various immune cells. **A** Cell distribution and model gene distribution in annotation cell based on the scRNA-sequencing in GSE162631 datasets from TISCH2 database. **B** SOCS3 expression patterns in spatial transcriptome analysis. **C** Unsupervised clustering result, all the samples were divided into two groups. **D** Kaplan-Meier analysis. **E** Correlation between subtypes and risk score

scRNA-seq data implied differential expression of the six key genes across various immune cell types (Fig. 8A). Among which, SOCS3 was highly expressed in monocytes and macrophages. Subsequent spatial transcriptome data based on SORC database also revealed that SOCS3 was highly expressed in monocytes and macrophages (Fig. 8B). Subtype analysis divided all the GBM samples into two clusters: cluster 1 (69 samples) and cluster 2 (97 samples) (Fig. 8C); and cluster 2 possessed worse survival and contained more high-risk samples (Fig. 8D and E, *P* < 0.01).

## Discussion

Many crucial life activities are mediated by ubiquitin and ubiquitin-like alterations, and their dysfunction is also associated with pathological progression such as immunological dysfunction and tumor progression [42]. PTM of PD-1 has been identified as a promising target for cancer immunotherapy, influencing the anti-tumor immune response of T cells [11–13]. Hence, early identification and prediction of diagnosis promote better survival and treatment efficacy. In our study, through WGCNA and machine learning algorithms, six SUN modified immunotherapy resistance genes were confirmed to involve in GBM prognosis: PLK2, CDC73, PSMC2, SOCS3, ETV4, and LMO7. Among which, CDC73, PSMC2, SOCS3, and ETV4 were substantially upregulated; whereas PLK2 and LMO7 were substantially downregulated in glioblastoma cells than that in control. Based on these six optimal genes, a prognostic risk model was generated, which exhibited excellent prognostic significance with AUC above 0.9. Through comparison of our risk score with ten previous published risk score, our risk score possessed best predicting ability in GBM, with better sensitivity and specificity in clinic application.

The six key genes exhibit significant role in GBM. The polo-like kinases (PLKs) constitute a family of serine-threonine kinases that play regulatory roles in various cellular processes. PLK2 is deliberated a potential inhibitory factor in tumors. PLK2 is down-regulated in GBM, and its upregulation links to poor prognosis [43], which is coincidence with our study. PLK2 might be a novel chemotherapy-resistant biomarker and therapeutic target in GBM. For example, Alafate et al. demonstrated that PLK2 inhibition could stimulate acquired resistance to TMZ through activation of PLK2/Notch axis [44]. Furthermore, the latest evidences indicates that PLK2 is also involved in various PTM process, including phosphorylation and ubiquitination [45, 46]. Tan et al. demonstrated that DYRK1A-mediated phosphorylation of PLK2 regulates the proliferation and invasion of GBM cells [43]. Ge et al. demonstrated that PLK2 can inhibit oxidative stress through phosphorylating GSK3β in ischemia-reperfusion injury [46]. CDC73, full named cell division cycle 73, has been demonstrated to regulate Notch-induced T-cell leukemia cells [47] in various disorders. For example, in esophageal cancer, CDC73 acts as a tumor-promoting factor [48]. CDC73 was upregulated in esophageal cancer, and its downregulation effectively hinders the proliferation and growth of esophageal cancer cells. In parathyroid cancer, CDC73 is a tumor suppressor gene, and variant of CDC73 is related to risk of parathyroid carcinoma [49]. Nevertheless, the specific role of CDC73 in GBM have not been clarified in GBM. A recent study demonstrated that CDC73 involve in ubiquitin-proteasome degradation in hyperparathyroidism-jaw tumor syndrome [50]. This evidence implied that CDC73 might affect GBM progression through regulation of ubiquitination, and the underline mediatory role should be clarified in future. PSMC2 is an important gene in proteasome complex. A pan-cancer analysis has suggested that PSMC2 serves as a reliable prognostic biomarker for predicting the response to immunotherapy [51]. Elevated expression of PSMC2 has been observed in gliomas and is associated with a poor prognosis for patients with this disease [52]; inhibition of PSMC2 can inhibit the cancer progression and drug resistance through regulation of immune microenvironment and cell autophagy [53, 54]. Evidence from other cancers also indicated the positive role of PSMC2 knockdown in tumor progression [55, 56]. SOCS3 represents a promising target for the treatment of metabolic disorders [57]. In GBM, SOCS3 is reported to be related to chemotherapy radiotherapy resistance acquisition [58, 59]. Overexpression of SOCS3 is found in GBM [58, 60], which is coincidence with our results. SOCS3 mainly regulate the GBM drug sensitivity through JAK/STAT phosphorylation signaling [60–62]. Furthermore, SOCS3 exhibits as an onco-immunological biomarker in GBM, attributing to its immune regulation role [63]. Goswami et al. demonstrated that Kdm6b absence enhances antigen presentation and anti-PD1 efficacy in myeloid cells by inhibition of Socs3 [64]. Hence, SOCS3 might also be an important immunotherapy target in GBM. E-twenty-six-specific sequence variant transcription factor 4 (ETV4) has also been demonstrated to involve in tumor progressions. In hepatocellular carcinoma, ETV4 elevation facilitates tumor metastasis by upregulating PD-L1 [65]. In Multiple Myeloma, ETV4-dependent transcriptional plasticity can maintain MYC expression and is related to drug resistance [66]. In GBM, ETV4 is upregulated and its knockdown promote the autophagy and cell apoptosis trough inhibition of PI3K/AKT/mTOR signaling pathways [67]. Furthermore, the phosphorylation regulation roles of ETV4 has also been reported in several cancers [68, 69]. LIM domain only 7 (LMO7) gene played crucial roles in regulating cell growth, differentiation, protein localization, signal transduction, and intracellular protein complex assembly, establishing it as a hallmark gene in cancer [70]. Research has indicated an inverse relationship between LMO7 expression and the progression as well as prognosis of human lung adenocarcinoma [71]. In pancreatic ductal carcinoma, LMO7 is reported to regulate the T cell differentiation and chemotaxis and thus achieve the immune escape [72]. Nevertheless, its function in GBM remains unexplored. In summary, there is currently a dearth of evidence linking these six genes to ubiquitination, SUMOylation, and Neddylation in GBM. Our study identified CDC73, PSMC2, SOCS3, and ETV4 as pivotal SUN-modified genes that contribute to resistance to anti-PD-1 therapy. Notably, these genes were also observed to be overexpressed in GBM cells relative to microglia. Conversely, PLK2 and LMO7 were downregulated. Suggesting that these six genes are appropriate to develop a predictive model, and the excellent predictive performance of the resulting risk scoring model further supports this point.

In our investigation, high risk score indicated a worse survival. The AUC value of the risk score model was above 0.9, which indicated a relatively high predicting efficiency, which exhibited potential application ability in predicting the survival of patients with GBM in clinic. That is, a patient with a higher risk score is likely to have a lower probability of survival. Furthermore, our results proved that the risk score constructed by the above six key genes revealed remarkably correlation with clinic features like IDH mutation, TMZ therapy, and MGMTp methylation. This results further demonstrated the advantages of the risk score in predicting GBM. Furthermore, GBM are highly heterogenetic. We examined the correlation between risk score and gene mutation. The results displayed that PTEN and TP53 were the main mutation genes. Among which, TP53 is a critical gene in normal tumor growth and its mutation predicts cancer deteriorate and drug resistance [73, 74]. Moreover, although p53 is known to undergo SUMOylation, the precise function of this modification in oncogenesis remains elusive.

More importantly, we found elevated immune cell infiltration levels in high-risk GBM, like higher regulatory T cell and natural killer cells, indicating immune microenvironment alteration. Moreover, 25 drugs displayed different IC50 between the two different risk status, indicating different sensitivity of patients with GBM to different drugs. Higher IC50 of Rapamycin, CGP.60,474, and AZD6244 were found in low-risk score, indicating GBM in low-risk group exhibited greater sensitivity to drugs. A higher TIDE score was associated with high-risk status, suggesting an increased likelihood of immune escape and a suboptimal response to ICB. These results imply that risks core could indicate the immune status and guide the selection of clinical drugs for GBM. Finally, we estimated the expression levels of the key genes across various immune cell types. scRNA-seq and spatial transcriptome analysis demonstrated that SOCS3 was highly expressed in monocytes and macrophages. SOCS3 is reported to be a modulator of macrophage, which could drive macrophage inflammatory responses and modulate the efficiency of phagocytic processes [75], indicating that SOCS3 might affect tumor progression and drug sensitivity through mediate the macrophages and monocytes in GBM.

In summary, we identified six genes associated with SUN-modified anti-PD-1 resistance in GBM and constructed a promising prognostic model. Our prognostic risk score system exhibited superior performance compared to ten previously published signatures. However, our study has several limitations that warrant consideration. First, our data comes from multiple databases and online sources, which might increase the data diversity and complexity. Second, our whole investigations and analyses largely depend on retrospective data; and small sample size may also induce some deviation. Third, although RT-qPCR and WB was used to verify the gene and protein expression levels, these results provide only essential experimental confirmation of the bioinformatic findings, and future investigations are necessary to experimentally validate specific SUN modification types (e.g., ubiquitination, SUMOylation, and neddylation) of these key genes in GBM cells by using approaches such as co-immunoprecipitation or mass spectrometry. Fourth, since the WHO CNS5 classification defined GBM as IDH-wildtype, but current TCGA and CGGA databases do not include this classification standard. Therefore, our analysis based on all IDH-wt and IDH-mut may lead to certain limitations. Finally, although we attempted to incorporate key clinical variables such as KPS and MGMTp methylation status into the multivariate Cox regression analysis, the absence of complete and consistent data across the TCGA and CGGA datasets prevented their full integration. Specifically, MGMTp methylation status was unavailable in TCGA dataset, while KPS data was missing in the CGGA-325 and CGGA-693 cohorts. These incomplete clinical profiling may limit the comprehensive adjustment for known prognostic factors in glioblastoma. Future studies with more complete clinical annotations are needed to validate and refine our prognostic model.

## Conclusion

A six-gene prognostic model related to ubiquitination, SUMOylation, and neddylation and anti-PD-1 response is constructed in GBM, and the model exhibits excellent predictive properties in the clinical survival and drug sensitivity, which is benefit for accurate prediction and refined treatment in clinic. Future research should focus on exploring the crucial role and potential mechanism of the model and model genes in GBM.

## Supplementary Information

Supplementary Material 1.

Supplementary Material 2.

## Data Availability

Data is provided within the manuscript or supplementary information files.
