# Supplementary material for: Identification and validation of SUN modification-related anti-PD-1 immunotherapy-resistance signatures to predict prognosis and immune microenvironment status in glioblastoma
Source: BMC Cancer. 2025 Nov 29;26:7. doi: 10.1186/s12885-025-15345-9 (PMC12771811; doi:10.1186/s12885-025-15345-9)
Supplement: Supplementary file 1 — Supplementary Material 1. [file 12885_2025_15345_MOESM1_ESM.docx]

**Figure S1. The function analysis for the module genes in the Weighted gene co-expression network analysis (WGCNA).**

**
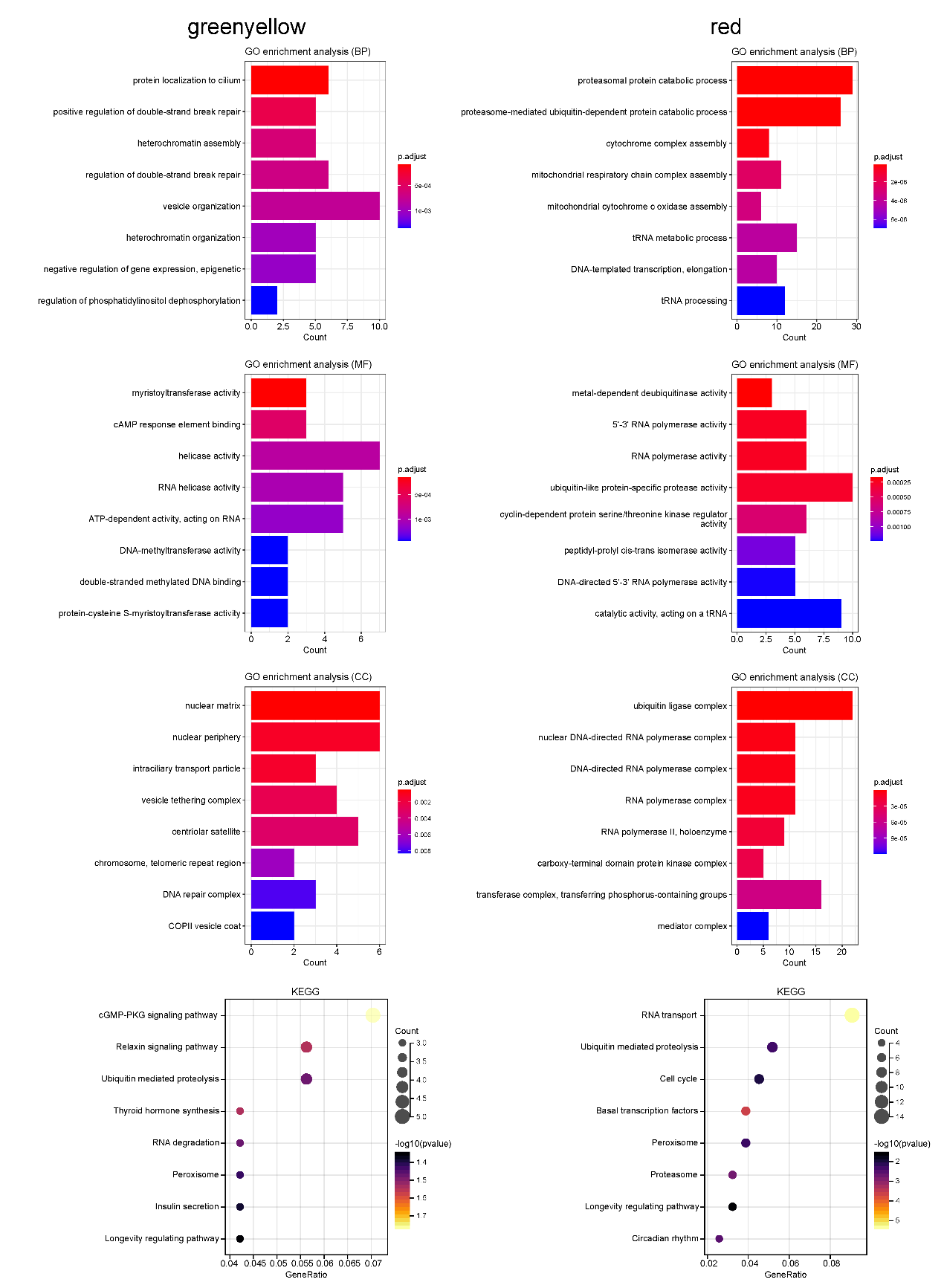
**

**Figure S2. Machine learning results.**

**
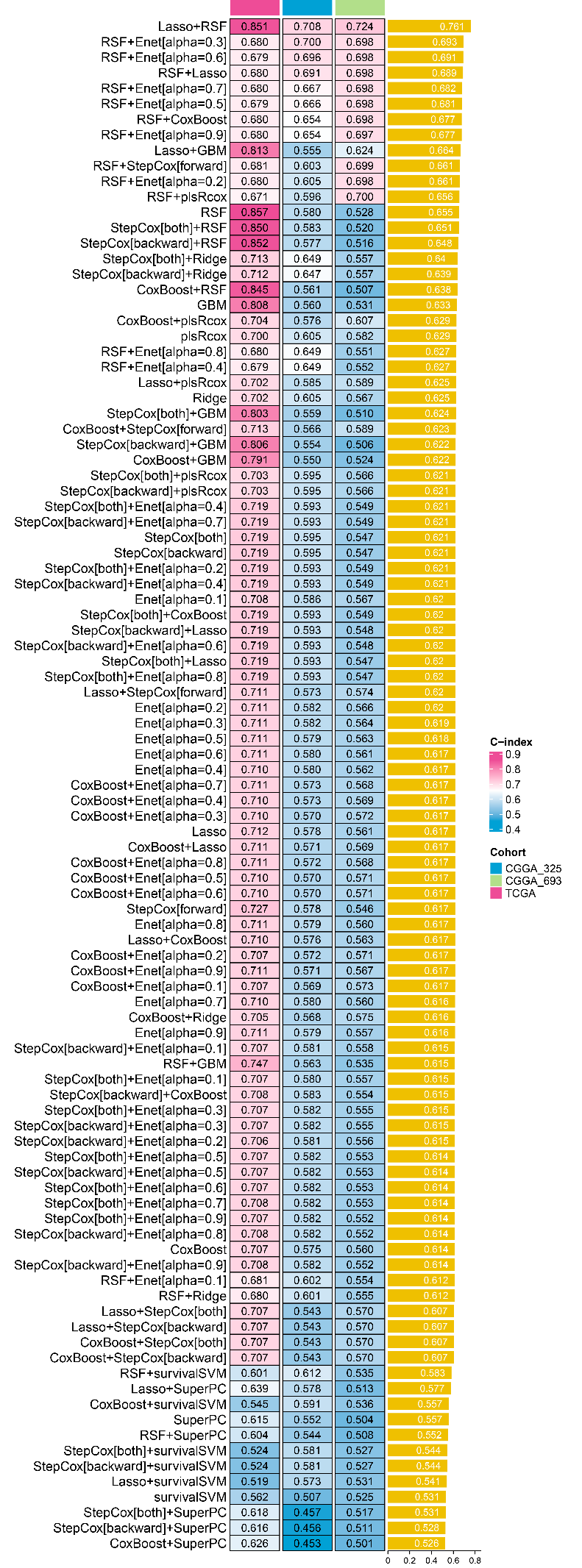
**

**Figure S3. The mutation patterns in high- and low risk groups. (A) The mutation patterns in high-risk group. (B) The mutation patterns in low-risk group.**

**
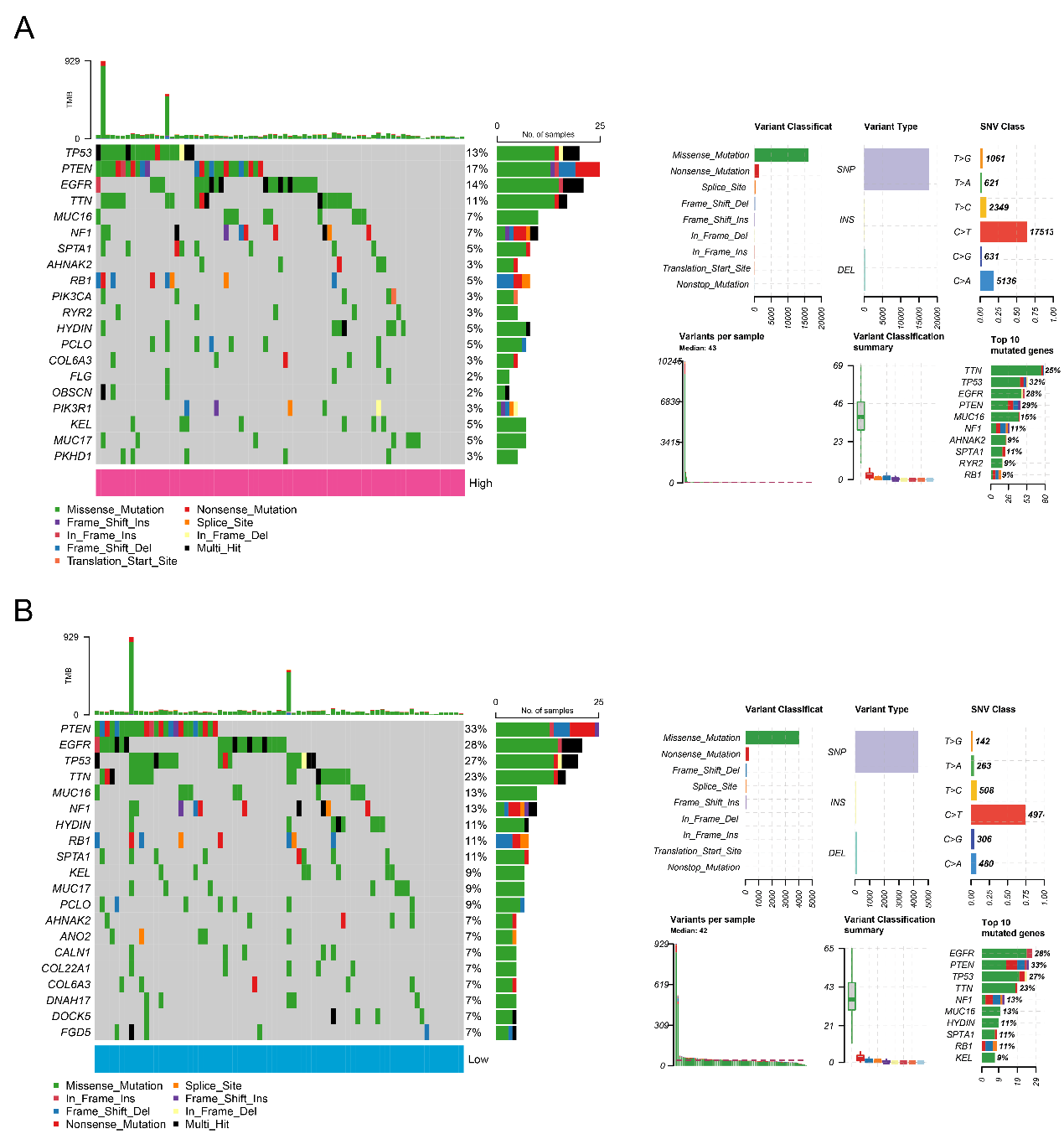
**
