# Supplementary figures and images for: Identification and validation of SUN modification-related anti-PD-1 immunotherapy-resistance signatures to predict prognosis and immune microenvironment status in glioblastoma
Source: BMC Cancer. 2025 Nov 29;26:7. doi: 10.1186/s12885-025-15345-9 (PMC12771811; doi:10.1186/s12885-025-15345-9)

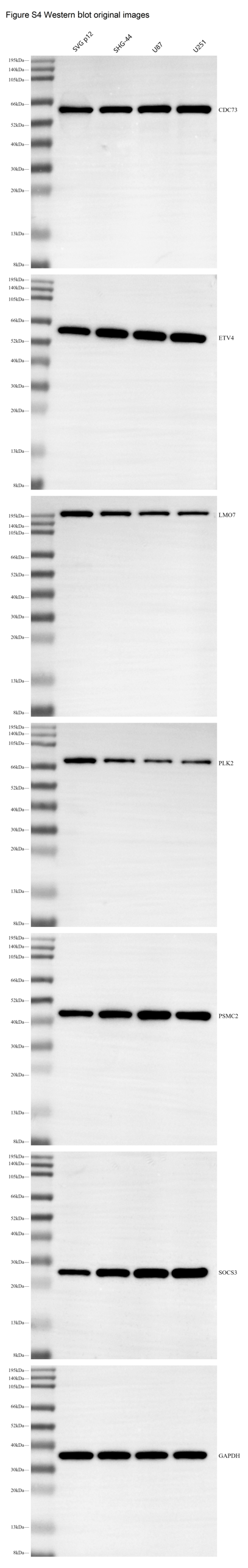

Supplement: Supplementary file 2 — Supplementary Material 2. [file 12885_2025_15345_MOESM2_ESM.tif]
